# Clinical Lectures on Diseases of the Eye

**Published:** 1864-11

**Authors:** E. L. Holmes

**Affiliations:** Chicago, Lecturer on Diseases of the Eye and Ear in Rush Medical College, and Surgeon of the Chicago Charitable Eye and Ear Infirmary


### CLINICAL LECTURES ON DISEASES OF THE EYE.

By E. L HOLMES, M. D., of Chicago,

*Lecturer on Diseases of the Eye and Ear in Rush Medical College, and Surgeon of the Chicago Charitable Eye and Ear Infirmary.*

### IRITIS.

*Gentlemen* :—From the number of cases of inflammation of the iris, which I have been able to bring before you during the present course, you may judge of the relative frequency of the disease. You will probably, in practice, meet one case of Iritis in every fifteen cases of diseases of the eye. If not on account of its frequency, certainly on account of the importance of the structures implicated, Iritis merits your careful study.

From whatever causes produced, the inflammatory action seems to commence in the spindle and star-shaped cells of the iris, but soon extends to the areolar tissue, the cells of the muscles and of the epithelium.

The swelling, redness, pain and sense of heat, experienced in the smallest pimple in the skin are a type of the symptoms which present themselves in iritis. The great vascularity, the extensive supply of nerves and the delicacy of structure explain the differences in the phenomena observed in iritis.

The objective symptoms of this disease are usually marked and readily detected, especially when the habit has been formed of examining the color and general aspect of the iris, the shape and movability of the pupil in different individuals in health. One, who has not formed this habit, will very often fail in his diagnosis. An error in diagnosis leads to an error in treatment, which is possibly more liable to do harm in iritis than in most other diseases. The most common mistake seems to be in confounding the vascularity in the early stages of iritis with that of conjunctivitis. Caustic astringents, so useful in the latter disease, are very dangerous in iritis.

One of the first symptoms of iritis is a sluggishness in the movements of the pupil when exposed to sudden changes of light and shade. This inactivity of the pupil is caused by the products of inflammation, pressing upon the nerves and muscles of the iris, impeding their action. Hence the pupil is fixed.

The fact which I have already mentioned, that the pupil is contracted by sleep and by irritation of the branches which the fifth pair of nerves send to the iris, will explain why the pupil is also contracted in iritis. The inflammatory action produces irritation of the nerves; this, with sleep, contracts the pupil. Consequently, as the disease progresses, the pupil becomes more and more diminished in size.

A discoloration of the iris with irregularities upon its surface, and especially upon its pupillary edge, is quite a constant symptom. The discoloration is produced by the extravasation from the cells and vessels into the substance of the iris. The formation of lymph is especially active at the pupillary edge of the iris, tending to unite the iris with the capsule of the lens. If the pupil is suffered to remain contracted it is liable to be completely filled with lymph, which not only unites the opposite edges of the pupil with each other, but also with the lens. In this case vision is almost totally destroyed. If the pupil is kept dilated without arresting the disease, the attachment between the lens and iris may take place, although the pupil itself and the central portion of the capsule of the lens may remain perfectly transparent.

Pain and redness, although perhaps always present, are symptoms exceedingly variable in degree. The pain may be but a slight uneasiness in or about the eye, or it may be agonizing beyond description. The redness is of a peculiar pink tint in the earlier stages of the disease, and is confined to a zone of fine radiating vessels of the subjunctival membrane around the cornea. Subsequently the conjunctiva itself may become congested and œdematous, when the characteristic color of iritis may disappear.

Obscuration of vision, produced by the products of inflammation, floating in the aqueous humor, is a marked subjective symptom. This cloudiness of the aqueous humor can be readily detected in ordinary cases. It requires, however, some experience in observation to distinguish it from certain cloudy appearances in the cornea when inflamed.

Pus is sometimes formed by iritis and thrown into the anterior chamber. Less frequently, perhaps, the rupture of a minute blood-vessel of the iris causes the presence of blood in the aqueous humor.

The causes of iritis are principally mechanical injuries, whether from accident or surgical operations, a syphilitic, scrofulous or rheumatic diathesis. With these predisposing causes, sudden changes of temperature, strong light, imprudent use of the eyes may become the active cause. Measles, typhoid fever and the absorption of pus into the circulation have been in rarer instances the apparent cause of the disease.

The termination of iritis is usually as follows: If the treatment has been skillful, all the tissues of the eye may escape uninjured. Even pus and blood may be absorbed from the anterior chamber and the lymph deposited upon the edges of the pupil (iris) and capsule of the lens may disappear.

The disease may run quite a rapid course, extending to the choroid and other tissues, and soon destroy the usefulness of the eye. You should remember that very often in proportion to the extent of the adhesions between the iris and lens will be the danger of relapse and ultimate loss of vision. Repeated attacks of iritis, after adhesions have been formed, seem inevitably to lead to choroiditis and loss of vision.

The substance of the iris may be filled to such an extent with the the products of inflammation, and the nutrition of the muscles and nerves so impeded as to destroy the true structure of the iris in the same way, as we have already seen, that the conjunctiva may become a thin, parchment-like membrane in zeropythalmia.

The course of few diseases, we believe, is so favorably modified by treatment as that of iritis. Pneumonia, dysentery and other similar diseases may recover without treatment and the organs implicated may be as healthy as before. I believe that inflammation of the iris, uninfluenced by treatment, rarely leaves vision as perfect as before the attack.

Omitting all theories and explanations, I shall allude but to a few of the most practical and important rules in the treatment of iritis. The following, I believe, are the three points of greatest importance: to keep the pupil dilated from the commencement of the attack by the frequent instillation of a strong solution of atropine; to confine the patient in a quiet room, with suitable temperature and little light; to allay pain by the use of anodynes. By a careful attention to these three points, you will find little difficulty in relieving uncomplicated cases of iritis. I usually employ a solution of the neutral sulphate of atropine, four grains to the ounce of water. A couple drops of this should be introduced between the lids every hour, more or less frequently according to the severity of the case. As an anodyne you may use morphine, cannabis indica, or codeia. The two latter are valuable agents in cases where morphine produces unpleasant effects. The doses should be sufficiently large to subdue the pain. Hot fomentations on the forehead and temple are of some assistance in reducing pain. Veratrum viride, aconite and gelsemine, if they do not tend to cure the disease, certainly palliate the symptoms, even where there is not much general fever. The diet should be light, and mild cathartics administered as required. Suitable constitutional treatment should, of course, be adopted in cases of complication with rheumatism, syphilis or scrofula.

I have never treated a case of iritis without prescribing mercury. You are aware that old authorities consider this remedy as all-important in iritis. Within the past few years, however, several oculists, especially Dr. H. W. Williams, of Boston, have shown that iritis can be successfully treated without mercury. Although this modification in the treatment is gaining favor, I think comparatively few distinguished oculists of different countries have discarded the use of this remedy. It should only be given in such doses as scarcely to effect the gums, care being taken to adapt the quantity to the susceptibility of the patient to its influence.

I have succeeded so well in treating iritis upon the plan above described, that I have not found reason to change it, even in omitting mercury. I am, however, more and more inclined to make the trial. The paper by Dr. Williams, and other shorter articles upon the subject by different writers, in medical journals, are worthy your consideration.

In cases of extensive adhesions between the iris and lens, with tendency to relapse, the most successful treatment is the removal of a portion of the iris, as in the operation for artificial pupil, which will be described in the following lecture.

The monograph of Graefe on chronic iritis, and the chapters on iritis in the works of Stellwag and Arlt, in addition to those in our best English text books should be carefully studied.

